# The use of 33 MHz ultra-high-frequency ultrasonography for the evaluation of sweat glands in the axilla with osmidrosis

**DOI:** 10.1371/journal.pone.0251600

**Published:** 2021-05-13

**Authors:** Akira Shinaoka, Ryuichi Nakahara, Masanori Saeki

**Affiliations:** 1 Department of Human Morphology, Okayama University Graduate School of Medicine, Dentistry and Pharmaceutical Science, Okayama, Japan; 2 Department of Plastic and Reconstructive Surgery, Okayama University Graduate School of Medicine, Dentistry and Pharmaceutical Science, Okayama, Japan; 3 Department of Orthopedic Surgery, Okayama University Graduate School of Medicine, Dentistry and Pharmaceutical Science, Okayama, Japan; 4 ViewClinic Momonosato, Tamano, Japan; King Faisal University, SAUDI ARABIA

## Abstract

**Background:**

This study aimed to assess the use of 33 MHz ultra-high-frequency ultrasonography (33MHz-UHFUS) for evaluating axillary sweat glands with osmidrosis in comparison with histological techniques. Axillary osmidrosis is a common problem in Asian societies, and the number and size of apocrine sweat glands have a strong relationship with osmidrosis severity. Currently, there are no methods to evaluate sweat gland distribution non-invasively.

**Methods:**

In this study, 35 skin specimens from 10 fresh human cadavers without osmidrosis and retrospective ultrasonographic images from 20 patients with osmidrosis were used. Skin specimens were embedded in paraffin, thinly sliced, and finally stained with hematoxylin and eosin. Histologically, the apocrine and eccrine glands were evaluated, and the top and bottom depths of follicles were measured from the skin surface. In 33 MHz ultrasonography images, the depths of sweat glands were measured, and the mean grey value was calculated using Image J.

**Results:**

Compared to histological data, 33MHz-UHFUS could be used to identify sweat glands as a hyperechoic structure between the dermis and fat layer. Furthermore, it could evaluate sweat gland distribution but could not distinguish between types of sweat glands.

**Conclusions:**

The distribution of sweat glands in the axilla can be non-invasively evaluated via 33MHz-UHFUS.

## Introduction

Axillary osmidrosis is a common problem in Asian societies, where people afflicted with such a malodor are shunned and considered to have a serious personal problem. Wet earwax, which has a strong relationship with osmidrosis, has a prevalence of 5–20% in East Asia and 97% in Europe or Africa [1].

Osmidrosis is a disorder characterized by excessive or abnormal body odor arising from apocrine sweat. Sweat itself does not smell; rather, skin bacteria (Corynebacteria) transform non-odoriferous precursors in sweat into a pungent odorant [2]. A specific aminoacylase in bacteria has been reported to catalyze a reaction that produces (E)-3-methylhex-2-enoic acid and (RS)-3-hydroxy-3-methylhexanoic acid from N-acyl-glutamine conjugates secreted into sweat in the axilla [3]. Other reports have claimed that since there are other odoriferous materials, the proportion of these components, such as lipophilic components, causes odor variance among individuals [4]. In addition, a histopathological study has shown that the number and size of apocrine glands have a strong relationship with osmidrosis [5].

A temporary solution for this distressing condition usually involves topical application of astringents, antiperspirants, or aluminum salt preparations [6]. Alternatively, local injection of botulinum toxin enables temporary control of axilla sweating and malodor by blocking the cholinergic innervation of the sweat glands [7]. Surgical excision of the skin and subcutaneous tissue [8], liposuction [9], sympathectomy [10], and electrodesiccation [11] reduce the volume of sweat glands and provide a more permanent solution for this problem.

A strong association of osmidrosis with a certain genotyping of the ABCCII gene has been reported [12]. However, in clinical practice, no definitive diagnostic criteria or objective measuring methods have been developed to characterize the severity of the odor, and whether an individual suffers from osmidrosis depends mainly on their assessment and/or on the examiner’s olfactory judgments. Therefore, new imaging methods that can evaluate gland volume and location are required for diagnosis and surgical settings.

Although ultra-high-frequency ultrasonography (UHFUS) over 20 MHz for the analysis of skin structures has become widespread, patients with sweat glands with osmidrosis have not yet been evaluated [13, 14]. In the present study, we compared findings from UHFUS at 33MHz (33MHz-UHFUS) with structures and distributions of sweat glands from histological studies to identify ultrasonographic imaging findings that indicate sweat glands and to determine the applicability of the former for structural analysis of sweat glands and clinical settings for osmidrosis.

## Materials and methods

### Histological study

Portions of axillary skin and subcutaneous tissue were excised from 10 Asian cadaver subjects without osmidrosis (Table 1). The cadavers were transported to the anatomy laboratory in the Department of Human Morphology, Okayama University, at the earliest opportunity. All donors agreed and provided written wills to donate their bodies for medical education and research. This cadaveric study was approved by the Ethical Committee at Okayama University Hospital (Ref. K1705-016). Written informed consent of the willed body donation program for medical education and research were signed previously by all donators. In addition, the form of consent by opt-out was taken.

**Table 1:** Cadaver and patient characteristics.

	Cadaver (n = 10)	Patients (n = 20)	P
Male, %	70%	50%	0.65
Age, years old (mean ± SD)	74.4 ± 13.1	19.0 ± 13.5	<0.001

SD: standard deviation.

From each of the 10 cadavers, a specimen measuring 3 cm × 2 cm was obtained from the midportion of the full-thickness hair-bearing axillary skin containing subcutaneous fat. After fixation in 4% formalin, these specimens were processed through a graded series of ethanol and embedded in paraffin. From 10 cadavers, 35 paraffin blocks were made and thinly sliced into 35 histological sections. After staining with hematoxylin and eosin, the sections were evaluated via light microscopy. From 35 histological sections, the top and bottom depths of 54 apocrine glands and 65 eccrine glands were measured from the skin surface.

### UHFUS study

Images of axillary skin were retrospectively taken using ultrasonography (Aplio i700; Canon, Tokyo, Japan) and a 33 MHz linear probe (PLI-3003BX; Canon, Tokyo, Japan) in 20 patients with osmidrosis (Table 1). Patients who underwent UHFUS by one examiner (M.S.) before and after injection of 0.5% lidocaine hydrochloride monohydrate (0.5% Xylocaine with epinephrine; Aspen Japan K.K., Tokyo, Japan) for radiofrequency ablation between January, 2019 and January, 2020 in the ViewClinic Momonosato were registered in this study. Patients underwent ultrasonography in a raised hand position under the same parameter settings (7mm focal depth, 86db 2D gain). Time gain compensation (TGC) was kept fixed at the central position throughout all the examinations. The patient study was approved by the Japan Medical Association Ethical Review Board (R2-4). The form of consent is opt-out because the study design is retrospective study. Imaging data were obtained and montaged with an Image Composite Editor, and the depth of glands and mean grey value were calculated using ImageJ by A.S. and R.N. [14]. From one patient, one image at the middle portion of the axilla was selected, and 20 images were registered. An 8 ×8 pixel square was used as the region of interest (ROI) for each region (dermis, root of hair, sweat gland, and fat).

### Statistical analysis

The mean grey value for each region was standardized with the grey value of the interstitium as the background. For continuous variables, we used the Kruskal-Wallis rank-sum test (among four groups) and Mann-Whitney U test (between two groups) with Bonferroni correction (for post hoc testing) to compare the background of patients (age in years), the mean grey value of tissues, and the distribution of sweat gland. Discrete variables (sex) are presented as percentages; comparisons were based on Fisher’s exact test. For the post-hoc test, statistical powers between each pair were calculated. Variables were considered significant when p<0.05. We performed all analyses in this study using EZR version2.6–1 [15].

## Results

In the histological study, 10 cadavers (7 male and 3 female) were used, and the mean age at death was 74.4±13.1 years old. In the ultrasonographic study, 20 patients (5 male and 5 female) were registered, and the mean age of ultrasonographic examination was 19.0±13.5 years old. Between the two study groups, age showed a statistically significant difference (P<0.001), but sex did not (P = 0.65).

**Fig 1:**
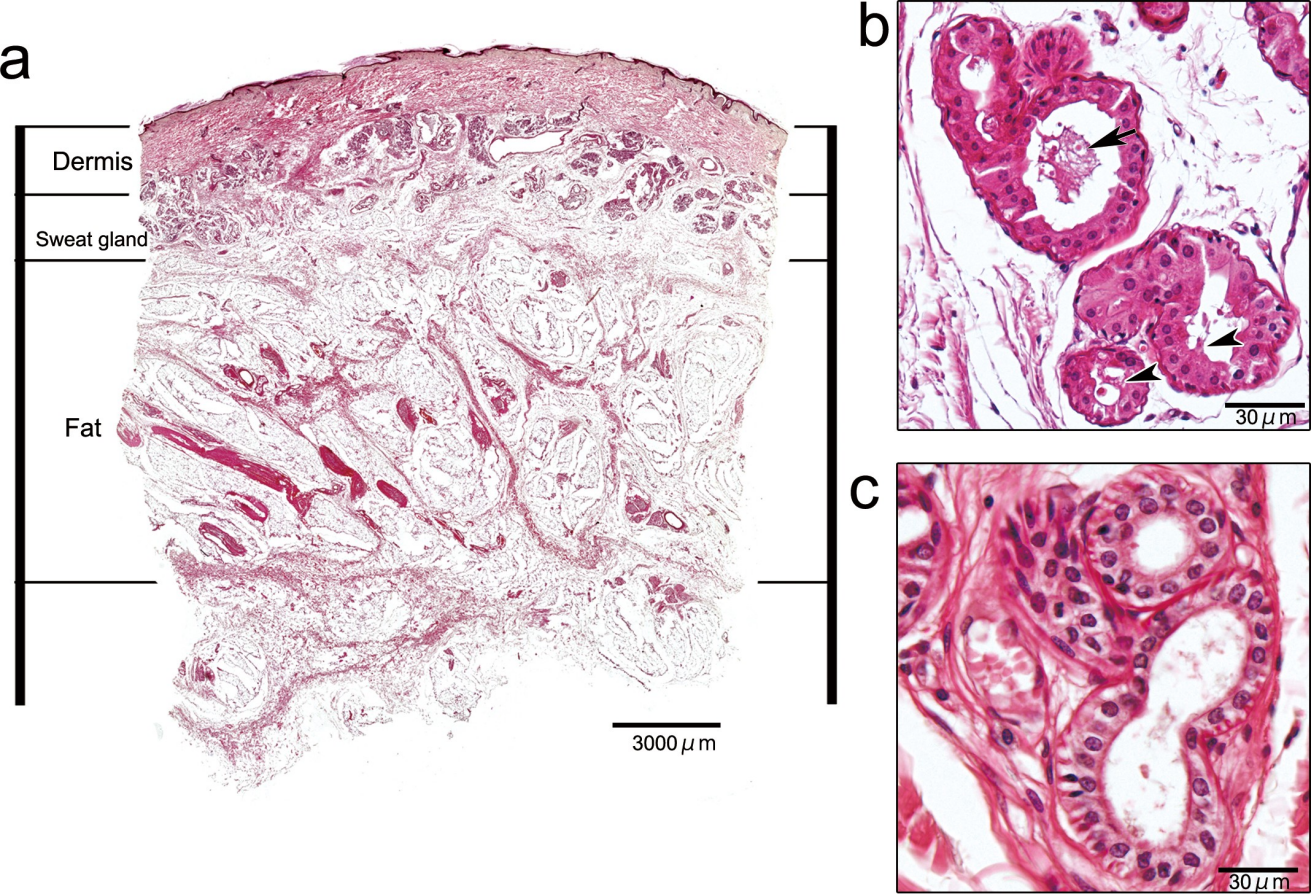
Histological image of the skin in the axilla. Sweat glands are located zonally between the dermis and the fat layer (left). The apocrine gland (top right) has secretory blebs (arrowheads) and secretory products (arrow) in the secretory coil. The secretory coil of the eccrine gland (bottom right) has two cell layers (inner: cuticular cell; outer: poroid cell) and is covered with myoepithelial cells.

**Fig 2:**
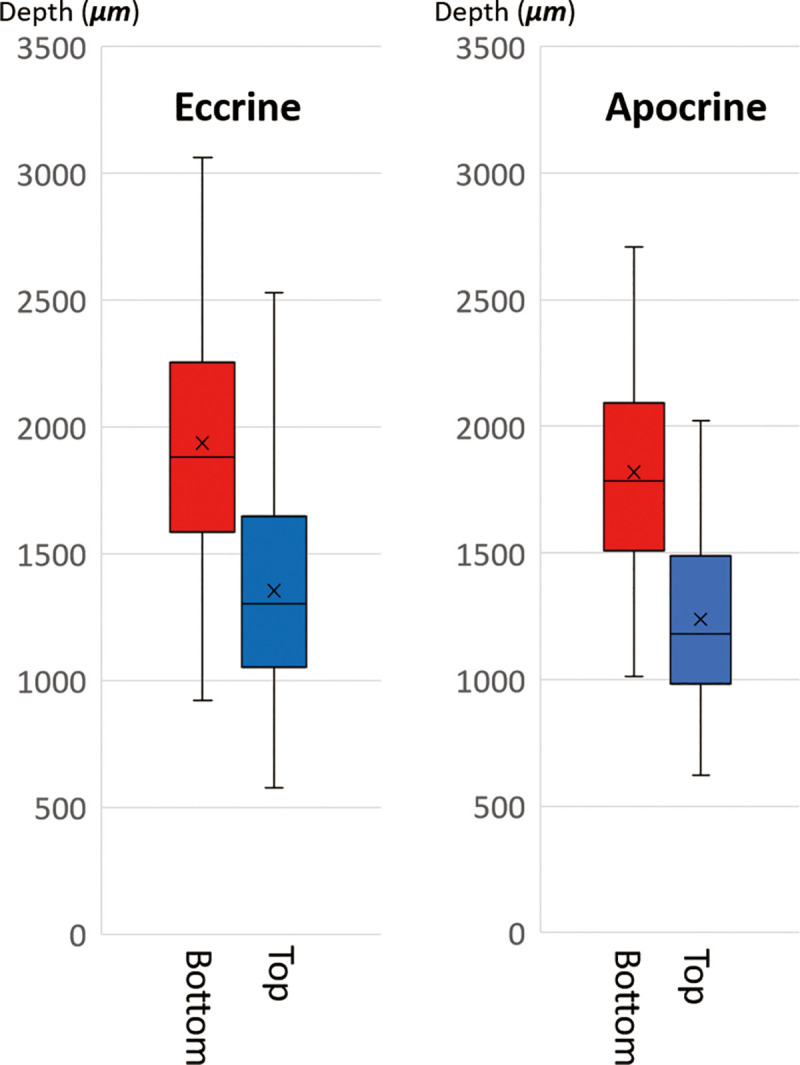
Depth of apocrine and eccrine glands from the skin surface in a histological study. The two secretory coils start at the boundary between the dermis and subcutaneous tissue, and are distributed to a depth of 1500–3000 μm. The distribution of these two glands is almost the same.

During the histological study of the cadaver specimens, sweat glands were observed as a zonal structure between the dermis and the fat layer. The apocrine and eccrine glands were distinguished from one another by referring to the apocrine glands’ histological features, such as secretory blebs and secretory products in the apocrine gland tubule (Fig 1). The vertical distribution of each gland from the skin surface is shown in Fig 2. The top of both types of glands was located at the boundary between the dermis and subcutaneous tissue, and the bottom depths of the apocrine and eccrine glands were 1819.9±398.3 μm and 1937.6±494.9 μm, respectively. These two glands were present as a mixed distribution and there is no significant difference between the distributions of the apocrine and eccrine glands.

**Fig 3:**
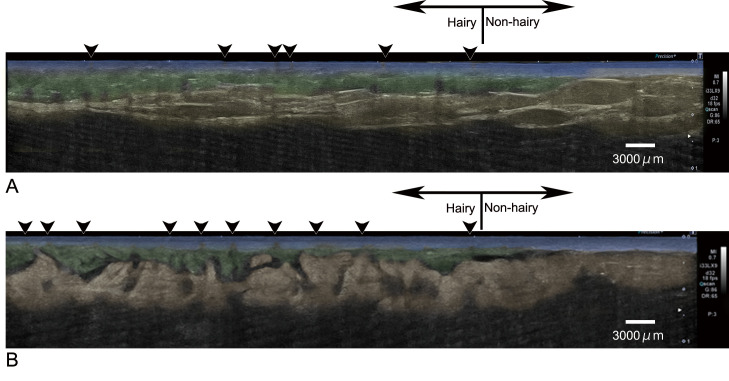
Sonographic image of skin in the axilla. Sonographic image before (A) and after (B) Lidocaine injection are shown with in color. The sweat gland layer (green) is observed as a hyperechoic area between the dermis (blue) and the fat layer (yellow) in the hairy region. Hair follicles (arrowheads) are observed as hypoechoic regions in the dermis. Subcutaneous injection of the solution created space between each tissue, making it easier to identify the sweat glands.

During the ultrasonographic study of the patients, the dermis, fat, and hair follicles were observed clearly (Fig 3 and S1 Video). Although it was impossible to distinguish between the two types of sweat glands, sweat glands could be observed as a hyperechoic region, especially after subcutaneous injection with lidocaine. Subdermal injection of the solution made it easier to identify sweat glands in the hypoechoic space at the interstitium. Furthermore, sonographic observation with moved skin made identification of the sweat gland easier because sweat glands moved separately from fat (S2 Video).

**Fig 4:**
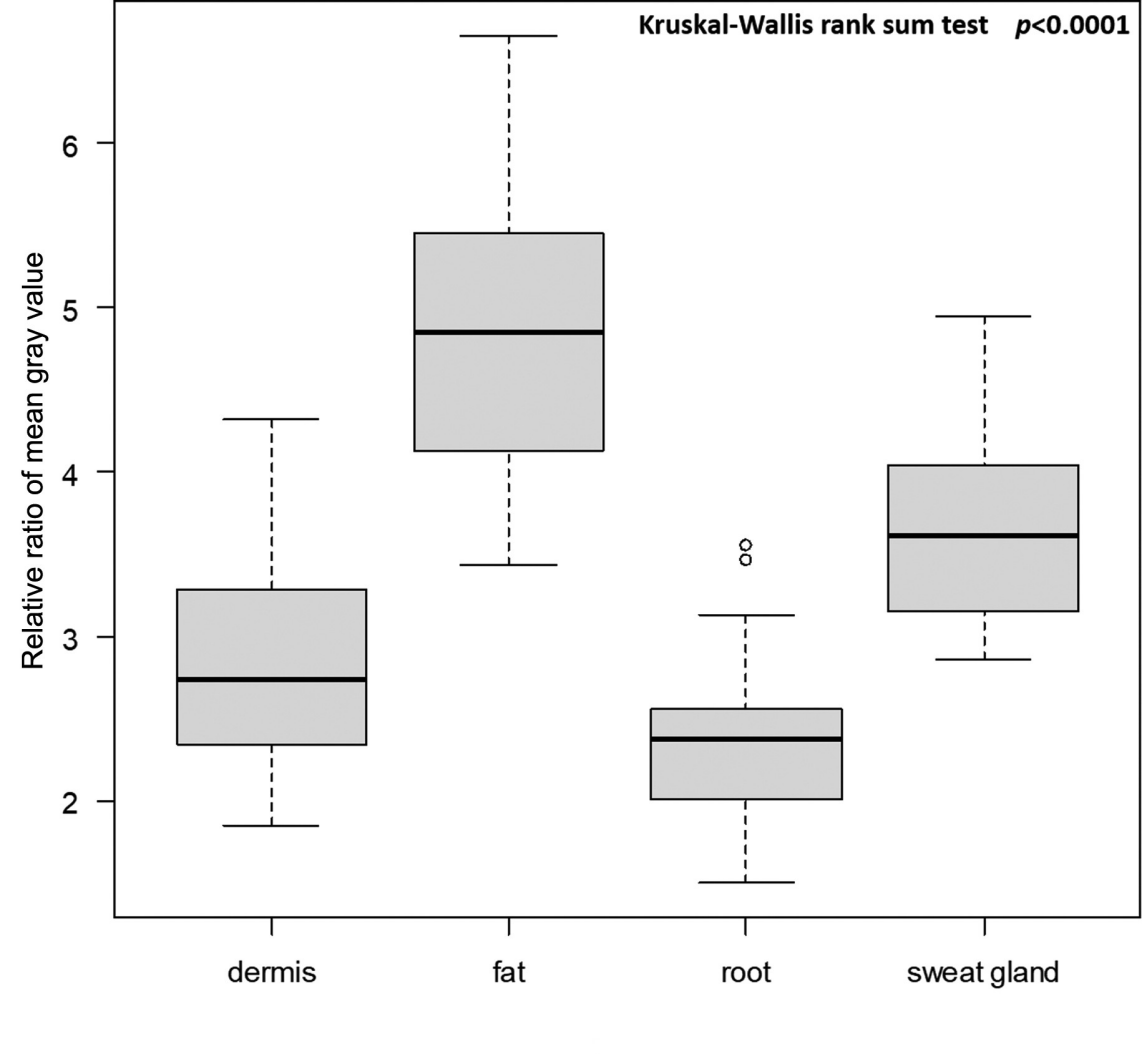
Relative ratios of mean grey values in the dermis, fat, hair root, and sweat glands compared to that of the interstitium. Twenty sonographic images after xylocaine injection in 20 patients were analyzed using Image J. Excepted for the comparison between the root and dermis, all pairs of P values are under 0.01.

The relative ratio of the mean grey value of sweat glands to interstitium was 3.68±0.64, which was higher than that of the dermis (2.85±0.67) and lower than that of fat (4.85±0.87). Except for the area between the dermis and root (P = 0.141), the mean grey value between each region was significantly different: sweat gland vs. dermis, P = 0.004; sweat gland vs. fat, P<0.001; sweat gland vs root, P<0.001; dermis vs fat, P<0.001; and fat vs root, P<0.001 (Fig 4). Post hoc testing showed that statistical powers between each pair were over 0.98: sweat gland vs dermis, 0.98; sweat gland vs fat, 0.99; and dermis vs fat, 1. Interobserver reliability between A.S. and R.N. was examined using the intraclass correlation coefficient (ICC). Interobserver reliability was graded as good for the depth of glands (ICC = 0.940) and mean grey value (ICC = 0.947)

**Fig 5:**
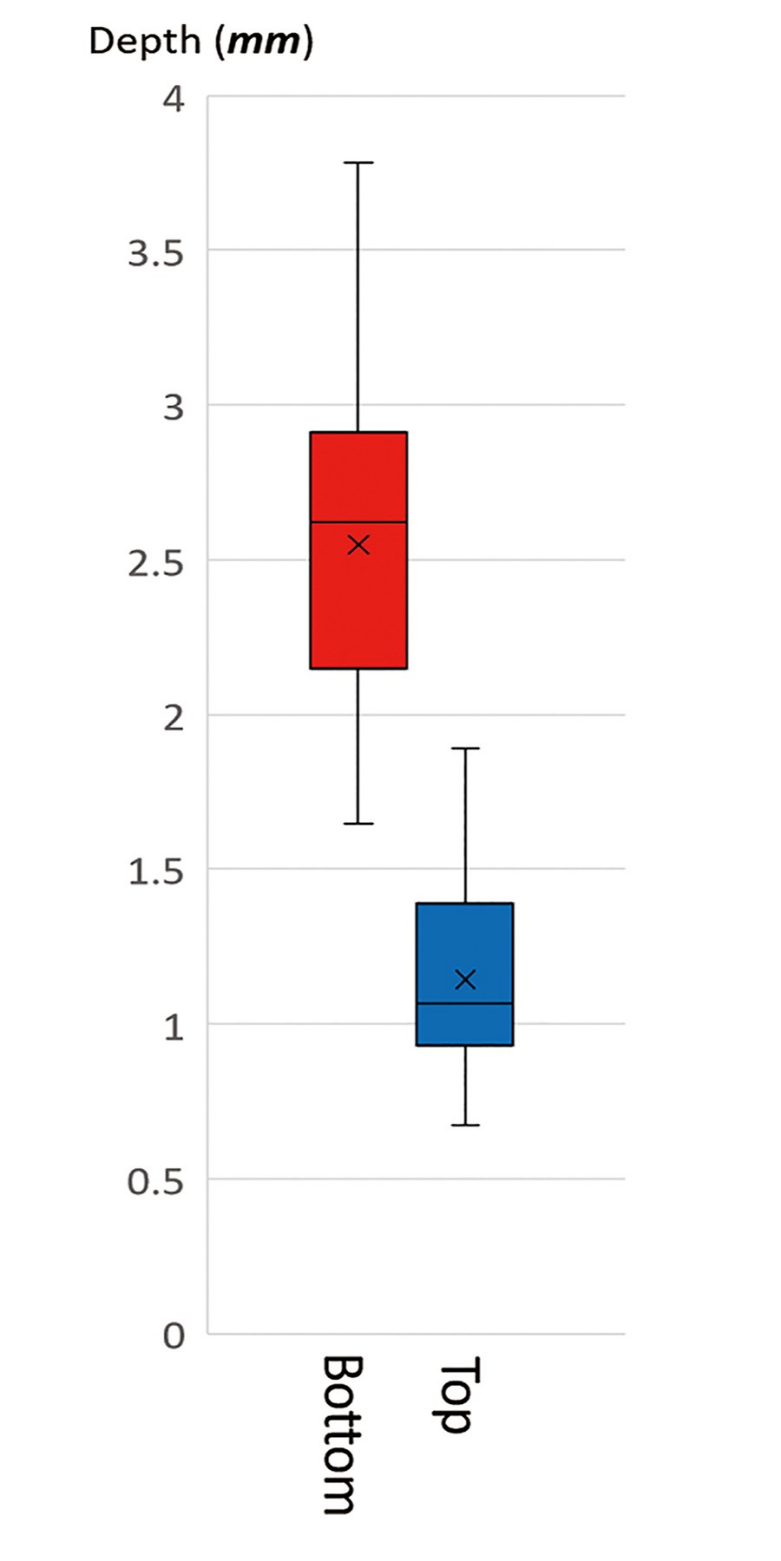
Depth of sweat glands from the skin surface in a ultrasonographic study. The sweat gland layer is located just on the dermis and distributed to a depth of 1500–3000 μm.

The vertical distribution of sweat glands from the skin surface is shown in Fig 5. Sweat glands were located up to the boundary between the dermis and subcutaneous tissue (1.14±0.30 mm), and the bottom of the glands was at a depth of 2.55±0.54 mm.

In the comparison between histological and ultrasonographic data, the distributions of bottom depth were significantly different (P<0.001), but the distributions of top depth were not significantly different (P = 0.0582).

## Discussion

The present study shows the possibilities and limitations of 30MHz-UHFUS for the evaluation of axillary sweat glands. A comparison with distribution data from histological studies demonstrated that the hyperechoic zonal layer between the dermis and fat layer was the sweat glands. The significant differences in bottom depth data between the histological and sonographic methods might be derived from differences in the osmidrosis symptoms and the age distribution of the subjects. While the histological subjects did not have osmidrosis symptoms, all ultrasonographic subjects were patients with osmidrosis. Furthermore, the development of apocrine sweat glands is completed in adolescence and begins to atrophy in middle adulthood. While the mean cadaver age was over 70 years old, patients were under 20 years old.

Evaluations of the volumes and distributions of sweat glands can certainly be informative for diagnosing and evaluating the severity criteria of osmidrosis because the development of sweat glands in the axilla is associated with osmidrosis symptoms [5]. However, high-frequency sonography is more limited compared to histological findings. Especially, it is not able to distinguish between apocrine and eccrine glands, although only apocrine glands have been associated with the severity of osmidrosis. But total evaluation without histological distinction would be sufficient for osmidrosis evaluation because a previous report showed that the eccrine gland size and the number of patients with osmidrosis were not significantly different from the control, and only the apocrine size and the number of patients of osmidrosis was larger than the control [5]. Thus, the total volume change of the sweat gland depends on apocrine sweat gland development; the age and total evaluation of the sweat gland would be sufficient for the evaluation of osmidrosis.

Furthermore, this technique may also be useful for pre-and post-operative evaluations of osmidrosis. In particular, as there tends to be a psychiatric component to osmidrosis, such as olfactory reference syndrome [16], quantitative visualization of sweat glands will provide insight into the true malodor condition of a patient and facilitate psychiatric rehabilitation after surgical excision.

UHFUS at a frequency greater than 70 MHz, although costly, has been shown to be useful for describing tissue at higher resolutions [17]. In particular, it was already reported that UHFUS could provide a better understanding of hidradenitis suppurativa, which was an inflammatory condition of sweat glands [18]. After various types and severities of osmidrosis are evaluated in the future, UHFUS could become a new technique for diagnosing and evaluating severity in patients with osmidrosis.

## Conclusions

33MHz-UHFUS can be used to evaluate the distribution of sweat glands in the axilla non-invasively. Furthermore, it has strong potential for the diagnosis and evaluation of severity in patients with osmidrosis.

## Supporting information

S1 Video Sonographic video of skin in the axilla. The sweat gland layer (green) is observed as a hyperechoic area between the dermis (blue) and the fat layer (yellow) in the hairy region.
(MP4)

S2 Video Sonographic video of sweat glands with moved skin. Sweat gland moved with skin, but fat layer moved separately from skin.
(WMV)

## References

[1] Sakai S, Imai K, Ogawa T, Iwaoka H. Japanese map of the earwax gene frequency: a nationwide collaborative study by Super Science High School Consortium. J Hum Genet. 2009;54:499–503. 10.1038/jhg.2009.62 19644513

[2] Emter R, Natsch A. The sequential action of a dipeptidase and a beta-lyase is required for the release of the human body odorant 3-methyl-3-sulfanylhexan-1-ol from a secreted Cys-Gly-(S) conjugate by Corynebacteria. J Biol Chem. 2008;283:20645–20652. 10.1074/jbc.M800730200 18515361 PMC3258934

[3] Natsch A, Gfeller H, Gygax P, Schmid J, Acuna G. A specific bacterial aminoacylase cleaves odorant precursors secreted in the human axilla. J Biol Chem. 2003;278:5718–5727. 10.1074/jbc.M210142200 12468539

[4] Natsch A, Derrer S, Flachsmann F, Schmid J. A broad diversity of volatile carboxylic acids, released by a bacterial aminoacylase from axilla secretions, as candidate molecules for the determination of human-body odor type. Chem Biodivers. 2006;3:1–20. 10.1002/cbdv.200690015 17193210

[5] Bang YH, Kim JH, Paik SW, Park SH, Jackson IT, Lebeda R. Histopathology of apocrine bromhidrosis. Plast Reconstr Surg. 1996;98:288–292. 10.1097/00006534-199608000-00012 8764717

[6] Inaba M. Human body odor: etiology, treatment, and related factors. New York: Springer; 1992.

[7] Bushara KO, Park DM, Jones JC, Schutta HS. Botulinum toxin—a possible new treatment for axillary hyperhidrosis. Clin Exp Dermatol. 1996;21:276–278. 10.1111/j.1365-2230.1996.tb00093.x 8959898

[8] Born G. Surgical treatment of axillary osmidrosis. Plast Reconstr Surg. 1995;96:1753. 10.1097/00006534-199512000-00061 7480315

[9] Ou LF, Yan RS, Chen IC, Tang YW. Treatment of axillary bromhidrosis with superficial liposuction. Plast Reconstr Surg. 1998;102:1479–1485. 10.1097/00006534-199810000-00021 9774001

[10] Hsu CP, Shia SE, Hsia JY, Chuang CY, Chen CY. Experiences in thoracoscopic sympathectomy for axillary hyperhidrosis and osmidrosis: focusing on the extent of sympathectomy. Arch Surg. 2001;136:1115–1117. 10.1001/archsurg.136.10.1115 11585501

[11] Lee JC, Kuo HW, Chen CH, Juan WH, Hong HS, Yang CH. Treatment for axillary osmidrosis with suction-assisted cartilage shaver. Br J Plast Surg. 2005;58:223–227. 10.1016/j.bjps.2004.07.002 15710118

[12] Nakano M, Miwa N, Hirano A, Yoshiura K, Niikawa N. A strong association of axillary osmidrosis with the wet earwax type determined by genotyping of the ABCC11 gene. BMC Genet. 2009;10:42. 10.1186/1471-2156-10-42 19650936 PMC2731057

[13] Bhatta AK, Keyal U, Liu Y. Application of high frequency ultrasound in dermatology. Discov Med. 2018;26:237–242. 30695672

[14] Rasband, W.S., ImageJ, U. S. National Institutes of Health, Bethesda, Maryland, USA, http://rsb.info.nih.gov/ij/, 1997–2007.

[15] Kanda Y. Investigation of the freely available easy-to-use software ’EZR’ for medical statistics. Bone Marrow Transplant. 2013;48:452–458. 10.1038/bmt.2012.244 23208313 PMC3590441

[16] Katharine A. Phillips M.D. and William Menard B.A. Olfactory Reference Syndrome: Demographic and Clinical Features of Imagined Body Odor. Gen Hosp Psychiatry. 2011;33: 398–406. 10.1016/j.genhosppsych.2011.04.004 21762838 PMC3139109

[17] Wortsman X, Carreño L, Wortsman CF, Poniachik R, Pizarro K, Morales C et al. Ultrasound Characteristics of the Hair Follicles and Tracts, Sebaceous Glands, Montgomery Glands, Apocrine Glands, and Arrector Pili Muscles. J Ultrasound Med. 2019;38:1995–2004. 10.1002/jum.14888 30570163

[18] Oranges T, Vitali S, Benincasa B, Izzetti R, Lencioni R, Caramella D et al. Advanced evaluation of hidradenitis suppurativa with ultra-high frequency ultrasound: A promising tool for the diagnosis and monitoring of disease progression. Skin Res Technol. 2020;26:513–519. 10.1111/srt.12823 31825113

